# Acetylator status-guided rapid reintroduction of isoniazid in tuberculosis patients with drug-induced hepatotoxicity

**DOI:** 10.1016/j.jctube.2026.100622

**Published:** 2026-06-05

**Authors:** Cynthia van Arkel, Ralf Stemkens, Cécile Magis-Escurra, Wouter Hoefsloot, Neeltje Carpaij, Jakko van Ingen, Reinout van Crevel, Arjan van Laarhoven, Rob Aarnoutse

**Affiliations:** a Department of Pulmonary Diseases, Radboudumc Community for Infectious Diseases & Research Institute for Medical Innovation, Radboud University Medical Center, 6525, GA, Nijmegen, the Netherlands; b Department of Pharmacy, Pharmacology and Toxicology, Radboudumc Community for Infectious Diseases & Research Institute for Medical Innovation, Radboud University Medical Center, 6525, GA, Nijmegen, the Netherlands; c Department of Medical Microbiology, Radboudumc Community for Infectious Diseases & Research Institute for Medical Innovation, Radboud University Medical Center, 6525, GA, Nijmegen, the Netherlands; d Department of Internal Medicine, Radboudumc Community for Infectious Diseases & Research Institute for Medical Innovation, Radboud University Medical Center, 6525, GA, Nijmegen, the Netherlands

**Keywords:** Tuberculosis, Acetylator status, Isoniazid, Reintroduction, Drug induced hepatotoxicity

## Abstract

**Background:**

People who slowly metabolize isoniazid are at increased risk of drug-induced liver injury (DILI) and interruption of tuberculosis treatment. We hypothesized that immediate identification of slow acetylators among patients with DILI will facilitate rapid and safe reintroduction of isoniazid.

**Methods:**

Between 2021 and 2023, at our tuberculosis referral centre in The Netherlands, we evaluated an isoniazid acetylator status-guided rapid reintroduction of anti-tuberculous drugs in a cohort of patients with DILI. Patients' acetylator status was determined by phenotyping after a single dose of isoniazid, regardless of liver function abnormalities.

**Results:**

In total, 49 tuberculosis patients underwent Therapeutic Drug Monitoring (TDM) from 2021 to 2023, with 67% being slow acetylators as assessed by phenotyping. Out of 10 patients with DILI, 8 were slow acetylators, and the total exposure to isoniazid (AUC_0-24h_) inversely correlated with the days until onset of hepatotoxicity in those patients (R = -0.84, *p* = 0.002). Six out of ten patients with DILI received a single dose of isoniazid for phenotyping, five of whom appeared to be slow acetylators. These slow acetylators were restarted at a lower dose of isoniazid. The phenotyping strategy led to a shorter total treatment duration compared to current guidelines for DILI. However, we also propose an even more optimized strategy, in which fewer than 14 days of therapy are ultimately missed, which could further reduce the total treatment duration by more than a month.

**Conclusion:**

In tuberculosis patients with DILI, assessment of isoniazid acetylator status can guide rapid reintroduction of isoniazid. Combined with AUC_0-24h_ measurement through TDM, this approach supports appropriate dosing and may help shorten total treatment duration.

## 1 Introduction

Drug-induced liver injury (DILI) is a well-known and troublesome adverse effect during treatment of patients with tuberculosis (TB) that can lead to many and prolonged interruptions. There is wide variation in reported incidence rates (2–28%) due to differences in the definition of hepatotoxicity [1]. Upon administration of anti-TB drugs, asymptomatic elevations in transaminase levels are common, but hepatoxicity can also be symptomatic, and it can be fatal if not recognized and if treatment is not halted promptly.

Isoniazid plays a crucial role in the early bactericidal phase of tuberculosis treatment but is also one of the primary causes of hepatotoxicity, dependent on the so called acetylator status of patients. Isoniazid-induced liver toxicity typically occurs early in treatment, whereas pyrazinamide-related toxicity tends to arise later, and rifampicin is primarily associated with a cholestatic pattern, marked by increases in serum bilirubin and alkaline phosphatase levels [2].

Genetic polymorphisms in N- acetyltransferase 2 (NAT2), an enzyme that metabolizes isoniazid, determine the acetylator status. There are homozygous fast (rapid), heterozygous fast (or intermediate), and slow acetylators [3]. Fast acetylators show lower exposure to isoniazid in plasma/serum, while slow acetylators have higher than average isoniazid concentrations. The acetylator status can be assessed genotypically or phenotypically. The phenotypic assessment involves the determination of the plasma (or serum) half-life of isoniazid or the assessment of the concentration ratio (or metabolic ratio) of the metabolite acetyl-isoniazid to isoniazid in plasma at a fixed time after dosing [4]. Studies suggest that slow acetylators are at an increased risk for hepatotoxicity, whereas fast acetylators are more likely than slow acetylators to have microbiological failure and relapse [4], [5], [6]. Indeed, a randomized clinical trial has shown that adjusting isoniazid dosage based on acetylator status reduces hepatotoxicity and early treatment failure [7].

In case of DILI, treatment with all anti-TB drugs is temporarily stopped. The reintroduction of anti-TB drugs is delayed until liver enzymes have decreased [2] and this may lead to long interruptions and a need for prolonging treatment. We hypothesized that, by identifying the causative drug — particularly isoniazid, by using phenotyping after a single dose of this drug — more quickly, this process can be accelerated. This would allow for an earlier reintroduction of other anti-TB drugs, shortening the conventional, prolonged reintroduction strategy and treatment duration.

## 2 Methods

### 2.1 Setting and TDM practice

Radboud university medical center (Radboudumc, Nijmegen) serves as a tertiary TB referral hospital in The Netherlands, providing care for patients with severe or complex TB disease, co-morbidities such as HIV infection, and drug-resistant TB. To optimize treatment and minimize the risk of failure or adverse effects, Therapeutic Drug Monitoring (TDM, i.e. the individualization of drug doses based on plasma concentration measurements) is implemented routinely to ensure appropriate drug dosing [8], [9]. Pharmacokinetic sampling is performed after a minimum of two weeks of treatment, once steady-state concentrations of most anti-TB drugs are achieved. For first-line anti-TB drugs, plasma samples are collected at 2, 4, and 6 h after the dose [10]. All first-line anti-TB drug concentrations are measured simultaneously using validated LC-MS/MS assays that comply with EMA and FDA guidelines and that perform reliably in international proficiency testing [10], [11]. The total exposure (area under the plasma concentration versus time curve, AUC_0-24h_) to isoniazid and other TB drugs is estimated using limited sampling formulas, with doses adjusted to achieve the population average target [10]. As part of routine TDM, isoniazid acetylator status is also assessed after two weeks. A hospital pharmacist calculates the isoniazid and acetyl-isoniazid concentrations at 3 h post-dose, based on levels at 2 and 4 h using first-order pharmacokinetic formulas. An acetyl-isoniazid/isoniazid ratio of <1.5 indicates a slow acetylator, while a ratio > 1.5 suggests a fast acetylator (phenotyping, [12]).

### 2.2 Patients and acetylator status-guided rapid reintroduction

The assessment of acetylator status through phenotyping and the calculation of AUC_0-24h_ are also performed when drug toxicity, such as DILI (1), occurs. In this setting DILI was defined as alanine aminotransferase (ALT) or aspartate aminotransferase (AST) >5× the upper limit of normal (ULN), or ALT or AST 3-5× ULN with symptoms (upper abdominal pain, nausea, vomiting). A single dose (2) of isoniazid (300 mg) was given when symptoms had subsided, regardless of ALT or AST levels. When it was confirmed that patients were slow acetylators and AST and ALT values were between 2 and less than 5 times the upper limit of normal (ULN), we reintroduced rifampicin and ethambutol within a few days (3). Thereafter, isoniazid was reintroduced at a lower dose within seven days (4). Pyrazinamide was reintroduced last, within seven days of the start of isoniazid (5). In patients identified as fast acetylators, the timing of onset and other contributing factors to the development of DILI were assessed. The isoniazid dose was subsequently adjusted based on the AUC_0-24h_.

### 2.3 Evaluation and statistics

Participants who underwent isoniazid TDM between 2021 and 2024 were included from the Mycobacterial Cohort Study, a large ongoing prospective cohort study conducted at Radboudumc. This study was approved by the Medical Ethics Committee Oost-Nederland (METC Oost-Nederland, CMO NL2021–13231, not subject to the Medical Research Involving Human Subjects Act). All participants provided written informed consent at inclusion.

Patient characteristics were described for patients with DILI who had stopped their anti-TB drugs and in whom the acetylator status was assessed with phenotyping after a single dose of isoniazid. Sociodemographic data, laboratory results, acetylators status and isoniazid dosage were collected. Time to culture conversion was measured from the day the anti-TB drugs were first started, regardless of any interruptions.

Data analysis was conducted using SPSS 27.0.1.0. Descriptive statistics were calculated, with normally distributed data reported as mean with standard deviation and non-normally distributed data as median with interquartile range. Linear and logistic regression analysis assessed associations between independent variables on the one hand and continuous or categorical dependent variables on the other hand.

## 3 Results

### 3.1 Patients in whom TDM of isoniazid was performed

TDM for isoniazid was performed in 49 patients in our cohort between 2021 and 2024 (see flowchart in Supplementary Fig. 1). Two thirds (*n* = 33) were slow acetylators and one third (*n* = 16) were fast acetylators, based on phenotyping. The total exposure to isoniazid (AUC_0-24h_) was higher among slow acetylators than fast acetylators (33 vs 7 h*mg/L, *p* < 0.001). The TDM practice appeared to be very effective in achieving average population AUC_0-24h_ values after dose adjustments with a mean of 17 h*mg/L (IQR 14–21 h*mg/L). Patient characteristics and demographics according to acetylator status can be found in Supplementary Table 1.

### 3.2 Patients with DILI

**Supplementary Fig. 2:**
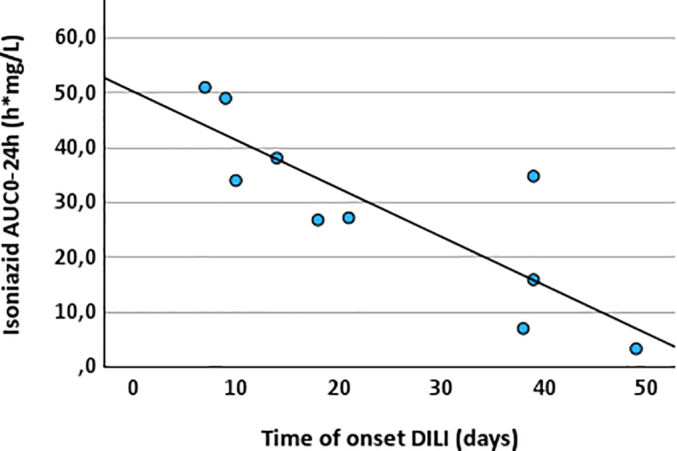
Correlation of exposure to isoniazid (AUC_0-24h_) and time to onset of DILI.*Spearman's R* *=* *-0.84 [CI -0.96 - -0.45], p 0.002.*

Among 10 out of 49 patients, DILI was diagnosed at some point during anti-TB treatment. Out of the 10 patients with DILI, 8 were slow acetylators and 2 were fast acetylators. There was an inverse correlation between the AUC_0-24h_ of isoniazid and the time to onset of hepatotoxicity, suggesting that a higher AUC_0-24h_ resulted in a shorter time to DILI onset (Spearman's R = -0.84 [CI -0.96 - -0.45], p 0.002) (Supplementary Fig. 2).

### 3.3 Patients with DILI and acetylator status-guided rapid reintroduction after a single-dose of isoniazid

**Fig. 1:**
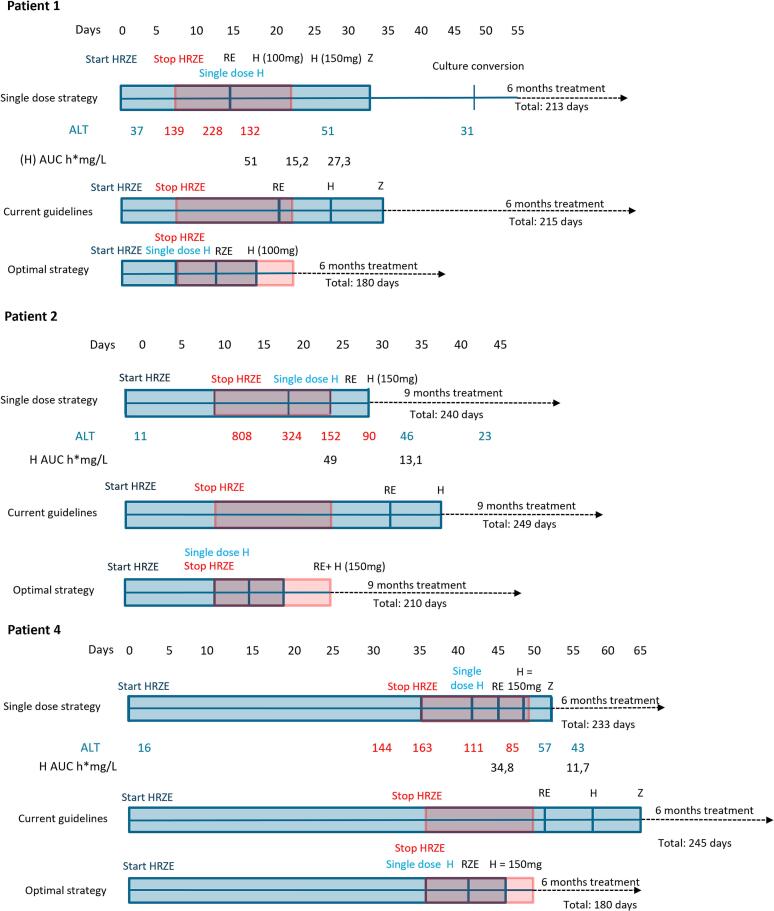
Illustration of various strategies to reintroduce anti-TB drugs after interruption due to DILI First row: timeline of the three most illustrative patients receiving a single dose of isoniazid, rechallenge with anti-TB drugs, and ALT levels (acetylator status-guided reintroduction strategy). Second row: scheme of the current guidelines is shown. Third row: the possible optimal strategy is presented. Patient 1 did indeed receive rifampicin and ethambutol (RE) simultaneously with single dose isoniazid (H), which slightly deviated from the protocol. As this was a retrospective study, small deviations from the established protocol occasionally occurred. Patient 2 had pyrazinamide-resistant *M. tuberculosis*; therefore, pyrazinamide was not reintroduced, and the patient was treated for 9 months with rifampicin, isoniazid, and ethambutol. The dotted arrow shows the total days of therapy for each strategy. In red ALT levels ≥2× times ULN when drugs normally would be reintroduced. In the red box, the 14 days of therapy are shown, after which the treatment is typically restarted for a period of 6 months if this timeframe is exceeded. Abbreviations: R = rifampicin, E = ethambutol, H = isoniazid, Z = pyrazinamide, ALT = alanine transaminase, AUC = area under the time-concentration curve 0-24H for isoniazid in h*mg/L. (For interpretation of the references to colour in this figure legend, the reader is referred to the web version of this article.)

In six of ten patients with DILI (1), the isoniazid acetylator status–guided reintroduction protocol was implemented (see flowchart in Supplementary Fig. 1, Table 1). Each patient received a single 300 mg isoniazid dose (2) with blood samples taken on the same day to assess the acetylator status regardless of the ALT level at that time (median ALT 111 U/L, range 22–324 U/L). Five patients (83%) were identified as slow acetylators with a mean AUC_0-24h_ of 40.5 (range 26.8–51.0) h*mg/L (far above the population average target of 15.2 h*mg/L [10]), and one was a fast acetylator (AUC_0-24h_ = 6 h*mg/L). After decrease of transaminases to below 5 times ULN, rifampicin and ethambutol (3) were typically restarted first (median 5 days after the single isoniazid dose), with isoniazid reintroduced at reduced doses (100–150 mg once daily) after a further median of 5 days(4). Dose-adjusted AUC_0-24h_ values for isoniazid at reintroduction in slow acetylators averaged 14.5 (range 11.6–15.7) h*mg/L, close to the population average target of 15.2 h*mg/L and acetylator status remained unchanged. Pyrazinamide was subsequently reintroduced (5) in all patients (median 5 days after isoniazid) (Fig. 1). No recurrence of DILI occurred, and culture conversion was achieved in all patients within 40–56 days (median 49 days). All six patients completed treatment without further hepatotoxicity or relapse during follow-up.

For each patient (Fig. 1) we also propose an optimal strategy in which less than 14 days of therapy are ultimately missed to show that the total treatment could be reduced by more than a month.

**Table 1:** Patient characteristics of six patients with DILI in whom acetylator status was assessed with metabolic phenotyping after a single-dose of 300 mg isoniazid.

	Sex	Age	Country of origin	Onset DILI (days)^2^	Max ALT (U/L)	ALT at time of single dose INH (U/L)	Acetylator status assessed by phenotyping	INH AUC_0-24h_ (h*mg/L	INH dose adjusted from 300 mg once daily	INH dose adjusted	AUC_0-24h_ after adjusted dose (h*mg/L)
1	Male	88	Indonesia	7	228	132	slow	51	yes	100	15.7
2	Female	42	Iran	9	808	324	slow	49	yes	150	13.1
3	Male	35	Eritrea	14	227	121	slow	38.1	yes	150	*
4	Male	59	Poland	39	163	111	slow	34.8	yes	150	11.7
5	Male	57	Suriname	18	84†	29	slow	26.8	yes	150	11.6
6	Male	33	Poland	49	119†	22	fast	3.3	yes	600	20.2

Abbreviations: DILI = Anti-TB drug-induced hepatotoxicity. INH = isoniazid. † AST > 5× ULN. ^⁎^no remeasurement took place. ^2^ DILI was defined as alanine aminotransferase (ALT) or aspartate aminotransferase (AST) >5× the upper limit of normal (ULN), or ALT or AST 3-5× ULN with symptoms (upper abdominal pain, nausea, vomiting).

## 4 Discussion

Phenotyping after a single-dose of isoniazid to assess acetylator status combined with TDM allowed us to retain isoniazid in the treatment regimen at a reduced dose in patients with a slow acetylator status, enabled a quicker reintroduction of other anti-TB drugs with limited prolonged treatment time for patients, and was successful without a relapse of DILI or signs of treatment failure. We also show that choosing a further optimization of this strategy, in which less than 14 days of therapy are ultimately missed, can further reduce the total treatment duration by more than a month.

**Supplementary Fig. 3:**
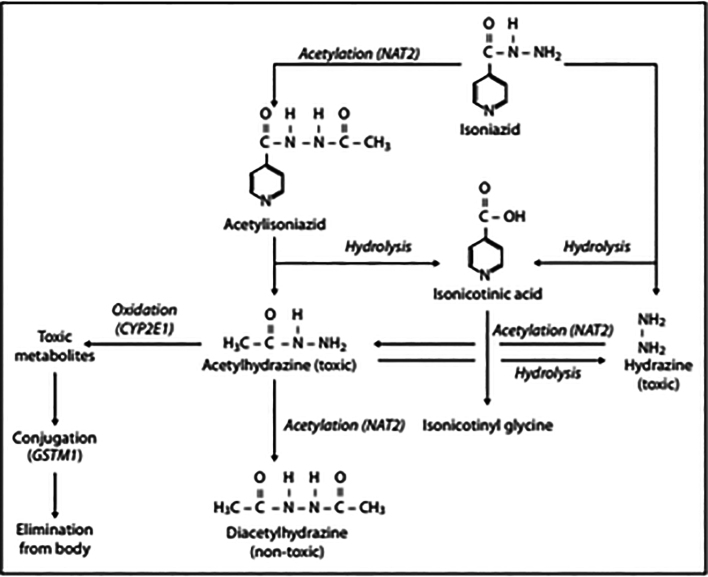
The metabolism of isoniazid [4], modified after Tostmann et al. [1].

Hepatotoxicity due to TB drugs is a well-known adverse effect. Up to 20% of individuals treated with isoniazid alone for tuberculosis infection (TBI) may experience low-grade, transient, asymptomatic transaminase elevation, mostly representing hepatic adaptation, which is a nonprogressive injury to hepatocyte mitochondria, cell membranes, or other structures [13]. Risk factors of DILI are slow acetylator status, advanced age, female sex, malnutrition, HIV, pre-existent liver disease and abnormal baseline aminotransferases [2], [14]. The mechanism by which isoniazid causes hepatoxicity is different from the other first-line anti-TB drugs. Genetic polymorphisms in the NAT2 gene result in homozygous fast, heterozygous fast (or intermediate) and slow acetylators. Slow acetylation results in higher exposures to isoniazid, but this is not thought to be the direct cause of hepatotoxicity. In slow acetylators, most isoniazid cannot be acetylated, but is directly hydrolysed to a toxic metabolite called hydrazine (see Supplementary Fig. 3). In contrast, in fast acetylators, isoniazid is rapidly converted to non-toxic diacetylhydrazine, which makes them less susceptible to DILI. In addition, it is argued that the intermediary product acetyl-hydrazine cannot be acetylated to non-toxic diacetylhydrazine in slow acetylators. In our current study, there seemed to be a relationship between the level of exposure (AUC_0-24h_) to isoniazid and the time to onset of hepatotoxicity in slow acetylators. Notably, four of our patients had an AUC_0-24h_ more than >30 h*mg/L. In our experience these patients appear to be at higher risk compared to those with an AUC_0-24h_ between 15 and 30 h*mg/L. One possible explanation is that slow acetylators with higher AUCs form more toxic metabolites, contributing to their increased risk.

Acetylator status can be assessed through genotyping or metabolic phenotyping. Genotyping requires complex equipment and involvement of experts to prevent pitfalls in methodology, terminology and specific mutations to be checked, dependent on the population studied [5], [15]. Metabolic phenotyping can be performed by determining the plasma or serum half-life of isoniazid or by measuring its plasma or serum concentration at a single time after oral administration. We chose to use the relatively simple phenotypic method of assessing the concentration ratio of acetyl-isoniazid to isoniazid in plasma at a fixed time, with an optimal sampling point at three hours after dosing [16], [17], [18], [19]. The alternative assessment of the elimination half-life of isoniazid formally requires the recording of a full pharmacokinetic curve, as a half-life needs to be assessed in the elimination phase of a drug. Still if no assay of acetyl-isoniazid is available, a rough estimation of the half-life of isoniazid could be made based on peak concentrations measured at 2 or 4 h post dose, with lower concentrations at 4 or 6 post dose. Patients with an isoniazid half-life of greater than 130 min (roughly 2 h) are classified as slow acetylators and those with a half-life of less than 130 min are fast acetylators [3], [12], [16]. A big advantage of simultaneously measuring the exact exposure to isoniazid (AUC_0-24h_) is that the appropriate dosage of this drug can be assessed. Disadvantages of phenotyping are the need for complex LC-MS/MS equipment, instability of isoniazid requiring immediate centrifugation and freezing of plasma at −80 °C, and the limited availability of acetyl-isoniazid as raw material [4]. Both phenotyping/TDM and genotyping are only available in a high-resource setting and are hardly accessible and costly in a low-resource setting where TB is most prevalent. Clearly, the development of an inexpensive point-of-care test (POCT) could significantly improve the availability and speed of determining acetylator status worldwide. This would allow high-risk patients, who require more intensive monitoring, to be identified in advance everywhere, and adjustments could potentially be made to dosing to reduce the risk of toxicity. It should be recognized that acetylation status is dependent of origin and therefore varies across different populations. About 50–60% of subjects from European (Caucasian), African and Indian origin are slow metabolizers, whereas a minority varying from 5 to 25% among populations from Asian origin (Chinese, Japanese, Eskimos) consists of slow metabolizers [4].

Current American guidelines advise to stop all hepatoxic drugs if the serum bilirubin is ≥3 mg/dL or 88 umol/L or serum transaminases are >5 times the ULN (or, in individuals with symptoms of hepatitis, serum transaminases >3 times ULN). Once these concentrations return to <2 times the ULN, anti-TB drugs can be restarted individually [2], [20]. A study showed that reintroducing all medications at once could also be safe, although this is debated by others, who argue that the main advantage of a sequential reintroduction is that it allows to identify the cause of hepatotoxicity more quickly [20], [21], [22].

The association shown in our study between the earlier onset of hepatotoxicity at a higher AUC_0-24h_ of isoniazid can help identify the right patients in whom isoniazid is considered the culprit. One patient in this cohort was identified as a rapid acetylator. Interestingly, DILI developed at a later stage in this patient (after 49 days). In hindsight, looking at the timing of onset, the decision to evaluate the acetylator status with a single dose isoniazid for this patient was not directly indicated. However, TDM allowed for a higher isoniazid dosage despite the DILI, and alcohol use was identified as the cause of hepatotoxicity. This highlights the importance of considering other contributing factors to hepatotoxicity.

In this study, we showed that isoniazid could be safely administered as a single dose, even when ALT levels exceeded 2× ULN. In confirmed slow acetylators, where isoniazid seemed the likely cause of hepatotoxicity, rifampicin and ethambutol were reintroduced early—while ALT was still >2× but <5× ULN—followed by a reduced dose of isoniazid. Pyrazinamide was reintroduced about a week later. This approach shortened the total treatment duration by several days to a week. Typically, stepwise reintroduction causes >14 days of interruption, requiring a full 6-month restart per guidelines [20]. Although our single-dose approach reduced therapy by days, more than 14 days were often still missed. To improve this, we propose early acetylator status determination, simultaneous reintroduction of rifampicin, ethambutol and pyrazinamide when slow acetylator status is confirmed, and TDM-guided dosing to keep interruptions <14 days. This could reduce overall treatment duration by about a month (Fig. 1). Further research in the form of a prospective, possibly multi-institutional study is needed to determine the safest, most feasible, and optimal rechallenge scheme.

Although most studies identify pyrazinamide as the most hepatotoxic drug [23], [24], our study suggests that most patients with hepatotoxicity were slow acetylators for isoniazid. By restarting isoniazid in an adjusted dose, both isoniazid and pyrazinamide could be continued in the treatment regimen. Although treatment without isoniazid is possible, it results in a regimen with 3–4 drugs (9RZE or 6RHZ-FQ). Even though the same treatment duration can be applied when a fluoroquinolone (FQ) is added, the total pill burden is higher than with standard treatment (HR) in the continuation phase. An increased drug burden may lead to non-adherence and, potentially, to acquired drug resistance.

Limitations of our study include the small group of patients and, owing to the referral center setting, a highly selected and specific patient population. Therefore, not all patients were admitted to our facility at the time of DILI onset, resulting in variability in the timing of the single isoniazid dose among patients, and this prevented us from earlier rechallenge as is shown by the proposed optimal treatment strategy. Additionally, the retrospective nature of the study contributed to this variability.

In conclusion, for patients with DILI, phenotyping using a single dose of isoniazid can help to identify slow acetylator status. This allows isoniazid to be retained in the treatment regimen at an optimized dose, facilitates faster reintroduction of other anti-TB drugs, and—when combined with an even more effective treatment strategy—may significantly shorten the overall treatment duration.

**The following are the supplementary data related to this article.Supplementary Fig. 1:**
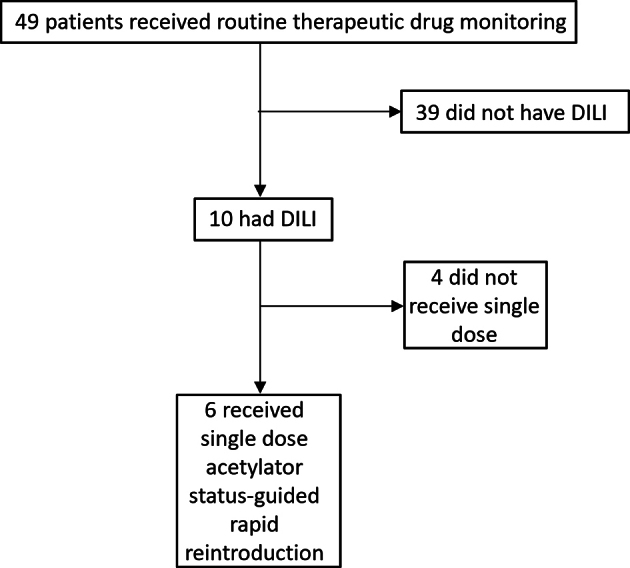
Flowchart of patients in the study.

Supplementary Table 1 Patient characteristics, laboratory and microbiological findings, toxicity, and therapeutic drug monitoring parameters for isoniazid.

## CRediT authorship contribution statement

**Cynthia van Arkel:** Writing – review & editing, Writing – original draft, Visualization, Validation, Supervision, Software, Resources, Project administration, Methodology, Investigation, Funding acquisition, Formal analysis, Data curation, Conceptualization. **Ralf Stemkens:** Writing – review & editing, Writing – original draft, Methodology. **Cécile Magis-Escurra:** Writing – review & editing. **Wouter Hoefsloot:** Writing – review & editing. **Neeltje Carpaij:** Writing – review & editing. **Jakko van Ingen:** Writing – review & editing. **Reinout van Crevel:** Writing – review & editing. **Arjan van Laarhoven:** Writing – review & editing, Writing – original draft, Supervision, Methodology, Formal analysis. **Rob Aarnoutse:** Writing – review & editing, Writing – original draft, Visualization, Validation, Supervision, Methodology, Formal analysis, Conceptualization.

## Funding

AvL was supported by a Clinical Fellowship of The 10.13039/501100001826Netherlands Organisation for Health Research and Development (ZonMw, 09032212110006).

## Declaration of competing interest

The authors declare the following financial interests/personal relationships which may be considered as potential competing interests:

Arjan van Laarhoven was supported by a Clinical Fellowship of The 10.13039/501100001826Netherlands Organisation for Health Research and Development (ZonMw, 09032212110006). The Wassink Hesp Foundation supported our Mycobacterial Cohort Study but had no further involvement in this research If there are other authors, they declare that they have no known competing financial interests or personal relationships that could have appeared to influence the work reported in this paper.
