# Supplementary material for: Acetylator status-guided rapid reintroduction of isoniazid in tuberculosis patients with drug-induced hepatotoxicity
Source: J Clin Tuberc Other Mycobact Dis. 2026 Jun 5;44:100622. doi: 10.1016/j.jctube.2026.100622 (PMC13266185; doi:10.1016/j.jctube.2026.100622)
Supplement: Supplementary Table 1 — Patient characteristics, laboratory and microbiological findings, toxicity, and therapeutic drug monitoring parameters for isoniazid. [file mmc4.docx]

**Supplementary table 1. Patient Characteristics, Laboratory and Microbiological Findings, Toxicity, and Therapeutic Drug Monitoring Parameters for Isoniazid.**

|  | Total group (N=49) | Slow acetylator (N=33) | Fast acetylator (N=16) |
| --- | --- | --- | --- |
| Demographics  Median age [IQR) - yr  Male sex – no. (%) | 44 [34-59]  33 (67%) | 44 [34-61]  21 (64%) | 46 [32-53]  12 (75%) |
| Origin – no. (%)  Sub-Saharan Africa  North Africa  Asia  Europe  Eastern Mediterranean  Americas | 16 (33%)  2 (4%)  4 (8%)  25 (51%)  1 (2%)  1 (2%) | 13 (39%)  1 (3%)  1 (3%)  16 (49%)  1 (3%)  1 (3%) | 3 (19%)  1 (6%)  3 (19%)  9 (56%)  0 (0%)  0 (0%) |
| Laboratory results  Baseline ALT [IQR] – U/liter Median Max ALT [IQR] – U/liter | 24 [14-38]  63 [33-123] | 26 [16-45]  57 [33-136] | 19 [11-32]  66 [26-115] |
| Microbiology  Median culture conversion*   [IQR] - (days) | 30 [21-53] | 28 [17-55] | 31 [23-47] |
| Toxicity  Drug induced liver injury – no.(%)  Median onset DILI [IQR] - days  Polyneuropathy – no. (%) | 10 (20%)  20 [10-39]  2 (4%) | 8 (24%)  16 [9-35]  1 (6%) | 2 (13%)  44 [38-44]  1 (6%) |
| Isoniazid TDM  Isoniazid dose adjusted – no. (%)  Median AUC0-24h baseline [IQR] - h*mg/L  Median AUC0-24h after dose adjustment [IQR] - h*mg/L | 34/48 (69%)  28 [9-38]  16 [13-19] | 21/33 (64%)  33 [28-39]  16 [13-18] | 13/15 (87%)  7 [6-9]  17 [14-21] |

*Abbreviations: DILI = drug induced liver injury. TDM = Therapeutic drug monitoring. ALT: alanine aminotransferase.* * Data available for 21 participants, 13 in the slow acetylator group and 9 in the fast acetylator group
